# Survey on the Acceptance of Ambient Sensors in Solo and Older Couple-Only Households in Japan

**DOI:** 10.3390/s23125522

**Published:** 2023-06-12

**Authors:** Toshiharu Igarashi, Misato Nihei

**Affiliations:** 1 Department of Human and Engineered Environmental Studies, The University of Tokyo, Kashiwanoha 5-1-5, Kashiwa 277-8563, Japan; 2 Institute of Gerontology, The University of Tokyo, 3-1, Hongo 7-chome, Bunkyo-ku, Tokyo 113-8654, Japan

**Keywords:** ambient sensors, elder care, online survey, assistive technology

## Abstract

For this study, an online survey was conducted to discover the preferences of older adults when they used sensors in their households, rather than the preferences of the researchers who developed them. The sample size was 400 Japanese community-dwelling people aged 65 years and older. The numbers of samples for men and women, household composition (single-person/couple-only household), and younger senior (younger than 74 years old) and older senior (older than 75 years old) were equally assigned. The survey results showed that “informational security” and “constancy of life” were considered more important than other factors when installing sensors. Furthermore, looking at the results regarding the type of sensors that face resistance, we found that both cameras and microphones were evaluated as facing slightly strong resistance, while doors/windows, temperature/humidity, CO_2_/gas/smoke, and water flow were evaluated as not facing such strong resistance. The elderly who are likely to need sensors in the future also have various attributes, and the introduction of ambient sensors in elderly households may be further advanced by recommending applications that are easy to introduce based on the attributes of the target population, rather than discussing all of them in general.

## 1. Introduction

It is known that in many developed countries the number of older people is increasing, and they desire to grow old in place, and the prevention of illness and disease through the development of healthy lifestyles and early detection and management can lead to a reduction in health care costs. Aging populations are accompanied by chronic diseases, and we are facing a worldwide shortage of medical personnel. It is known that preventing illness and disease by developing healthy lifestyles and early detection and management of such illnesses and diseases can lead to reductions in medical costs.

Lifestyles of the elderly are changing, and financial pressures on various medical support systems are increasing. In Japan, it used to be common for two or three families to live together. In recent years, the number of households consisting of a single person or an elderly couple has increased [1]. The current comprehensive community care system does not provide sufficient support due to the limitations of the system and human resources; according to an estimate of “nursing care personnel needed in the future” based on the 7th Long-Term Care Insurance Business Plan released by the Ministry of Health, Labor and Welfare in 2018, approximately 2.45 million nursing care personnel will be needed in 2025 [2]. Even if retirees are not included in this estimate, the number of caregivers in FY 2016 was approximately 1.9 million, which means that 550,000 new caregivers, or more than 60,000 per year, will need to be secured.

Although the government has taken various measures in the past, the shortage of caregivers has not been significantly resolved. Given the declining labor force in Japan, it is unlikely that the situation will improve dramatically in the future [3,4]. This shortage of caregivers makes it difficult to provide individualized care in order to achieve a healthy society. However, since previous studies have shown that Japanese elderly people prefer to live in the community and want to maintain that lifestyle, it is necessary to support people’s lives with appropriate technology [5,6,7].

The use of assistive technology can help the elderly become more independent. Ambient sensors installed in the living environment have shown promise [8,9]. This study was undertaken to analyze three aspects of assistive technology use among older people living in the community in Japan: factors that promote the installation of ambient sensors in their homes, the types of sensors that face resistance, and factors that are important in the selection of sensors.

## 2. Background

Assistive technology refers to any device, tool, or system that helps people with disabilities perform tasks in their daily lives, work, or education. These technologies are designed to assist individuals with visual, auditory, motor, cognitive, or communication impairments, enabling them to function independently and lead more fulfilling lives [10]. Many papers have reported that the installation of ambient sensors can not only protect life but also maintain or improve physical and mental health. A systematic review by Riitta et al. identified 944 papers, 16 of which reported effects of sensor intervention [11]. Older adults reported that their smart homes improved their sense of security, quality of daily life, and activities and provided information about the care they received. While the use of ambient sensors is becoming more common in nursing homes, the technology is not yet widespread in the homes of older adults in the community. This may be due, in part, to the fact that the intentions of the users of the sensors, the older adults living in the community, are not fully understood.

There are many different types of ambient sensors, and it is not possible to discuss them in general. Parisa et al. categorized ambient assisted living (AAL) tools by dividing them into environmental installation types and wearable/mobile types [12]. Among them, various types of ambient sensors are listed, including light, humidity, temperature, pressure, water flow, current, opening/closing, sound, and video sensors. A study by Li Na Lee et al. [13] investigated the factors needed for a pleasurable experience for older adults in smart residential environments. The results indicated that it is important for older adults to be aware of the perceived benefits of the technology. There are various possible applications. For example, the study by Kate et al. [14], which evaluated social robots, environmental sensors, and wearable sensors, provided adequate evidence of their effectiveness regarding health outcomes. In addition, factors related to mental health, such as “mental support”, and factors related to informational benefits for living an autonomous life, such as “personal disease management and medication”, were mentioned in the study by Li, Ruijiao et al. [15]. In a study by Kong et al. [16], in a review of mobile health for older adults living alone, data security and protection of user privacy were mentioned as factors that were important to users. This would suggest that informational security is also considered important. While some studies report that various aspects are important in the acceptance of environmental sensors, it is impractical to develop new sensors with equal emphasis on all the multiple factors.

Previous studies have been conducted not only on the development and evaluation of individual sensors but also on the level of acceptance of such sensors based on group interviews with a dozen or so older people [17,18]. The concept of saturation is applicable to group interviews, which have the advantage that qualitative interviews with subjects with homogeneous attributes can be conducted with fewer participants [19,20]. However, while group interviews can extract the necessary elements, it is difficult to numerically analyze the importance of each element. Therefore, a statistical survey with an appropriate sample size is considered necessary.

When conducting statistical surveys, it is expected that the factors considered to be important will vary depending on the demographics of the respondents. In a study by Tyge-F Kummer et al. [21], a survey was conducted regarding which factors facilitate the adoption of medication assistance systems among nurses. The adoption of novel systems was shown to be facilitated when the usefulness of the system being implemented was perceived via demonstrated results, positive image of the technology, and innovativeness of the individual. However, resistance to new systems increases as users grow older. The age difference of the elderly may be relevant in terms of the introduction of sensors at home. Even if the survey was to be limited to the elderly aged 65 and older, there are 17.5 million younger seniors (aged between 65 and 74 years) and 18.7 million older seniors (aged 75 years and older) in Japan; even within the bracket of older people, perceptions may differ [22]. A study by Morris et al. used the technology acceptance model (TAM) to examine the relationship between age and technology acceptance [23].

Factors other than age, such as self-efficacy, may also have an impact on a sensor’s acceptance. Nicolas et al. examined attitudes toward acceptance of VR headsets for fall prevention among older people. They hypothesized a cognitive model based on the results of factor analysis, arguing that the need for self-efficacy and avoidance of future interventions influenced awareness about potential benefits, which in turn led to willingness to use the new technology [24].

Thus far, no statistical survey studies have been conducted to compare factors that promote the installation of ambient sensors in the home, the types of sensors with resistance, and the factors that are important in the selection of sensors with 400 Japanese single/couple older adults. Therefore, in this study, we limited the type of sensor to ambient sensors, conducted a survey of the opinions of the target population on the acceptance of ambient sensors among single-person and elderly-couple households in Japan, and analyzed the basic attributes that led to the acceptance of each type of sensor. We also analyzed the possibility that age, self-efficacy, and actual higher-order life functions were related to the level of acceptance.

## 3. Methods

### 3.1. Determination of Sample Size

This study was conducted via means of an unscored online survey of 400 persons living alone or in households headed solely by an elderly couple aged 65 or older in Japan. According to the National Survey of Living Conditions in Japan, the number of elderly people living alone in Japan is 7.42 million, and the number of elderly couples living alone in Japan is 16.5 million (8.25 million households times the number of elderly couples), for a total of approximately 23.92 million [1]. The following Equation (1) was used to calculate the required sample size [25]. Using this formula, the calculation yields *n* = 384.16.

The sample size becomes even more appropriate when finite population correction (FPC) is applied; to apply FPC, the following Equation (2) is used. The corrected sample size is calculated to be $\left|n_{adj}\right|$ ≈ 384.99. In this case, the sample size after modification is almost the same as the sample size before modification. In this case, 400 was used to balance the gender/age/household composition (single/couple). This is above the required sample size of 385.

(1) $$n=\frac{Z^{2}\times p\times \left(1-p\right)}{E^{2}}$$

(2) $$n_{adj}=\frac{n}{1+\frac{n-1}{N}}$$

*n* = sample size
*N* = population size
*Z* = *Z* value at the confidence interval
*p* = Probability of occurrence of the evenet in the population
*E* = Tolerance (in this case, 0.05)

**Sample size:** 400 persons.

**Participants:** Japanese community-dwelling older persons aged 65 years and above (living alone or in a couple-only household).

**Allocation:** Equal numbers of men and women, pre- and post-elder (65–74 years/75 years and older), and single-person/married-couple-only household composition.

### 3.2. Questionnaire Items

A total of 57 items were included in the survey (Table 1). The items included questions on subjective health (1 item), self-efficacy (10 items), life functions (16 items), factors that promote the installation of ambient sensors in the home (8 items), types of sensors that face resistance (10 items), and factors that are important for the installation of sensors (12 items).

Subjective health assessment: The health status of the respondents may have had an impact when selecting a health application for the installation of sensors. Therefore, we added an item asking about the respondents’ subjective health evaluations. For the subjective health evaluation, based on the Cabinet Office’s “Survey on the Health of the Elderly” conducted in 2017 [26], the respondents were given the option to respond to the question “Please tell us your current subjective health status” on a 5-point scale from “1: Good” to “5: Not good”.

Self-efficacy: Scales measuring self-efficacy that are written in Japanese include the 10-item scale of Schwarzer et al. [27] and the 16-item scale of Sakano et al. [28]. In this study, we decided to use Schwarzer et al.’s 10-item scale in order to reduce the burden on respondents due to the overall increase in the number of items and based on its proven use in Japan.

Activity capacity index: In addition to subjective health evaluation, respondents’ actual life functions may also have an impact on the acceptance of a sensor. Various scales measure low-to-high life functions. The Barthel Index [29], Katz Index [30], and FIM (Functional Independence Measure) [31] measure lower-order life functions, while the Lawton Scale [32], AMPS (Assessment of Motor and Process Skills) [33], FAI (Frenchay Activities Index) [34], and DASC-21 [35] can measure higher-order life functions as well. However, the FAI is not suitable for online questionnaires because it requires an interview, and the number of items on the Lawton scale varies depending on whether respondents are men or women. The AMPS and DASC-21 are observational scales that require responses from caregivers or people living with the patient, not from the patient.

Since the older people living in the community included in this study were assumed to be those who did not need to be institutionalized and had no problems using PCs and smartphones, it was expected that the scales measuring presentational life functions would not make a difference. Therefore, we decided to use the JST version of the activity ability index developed by Suzuki et al., which is considered the most suitable for measuring activity ability in community-dwelling older people as per previous studies [36].

Types of sensors: We did not include wearable/mobile-type sensors in this study, but only sensors installed in the environment, in reference to the previous study by Parisa et al. that classified sensors [12].

Sensors installed on walls and ceilings, floors/mats, doors, and windows were included as those that acquired numerical information on human movement. Video and microphone sensors were also included as sensors that directly acquired information. The sensors used to detect abnormalities included body temperature sensors; temperature/humidity sensors; and CO_2_, gas, and smoke sensors. In addition, electricity and water-flow sensors were included as sensors to indirectly estimate living conditions.

Necessary elements for sensors: The key elements for sensor installation were developed with reference to QUEST [37], which consists of twelve items, eight of which related to the usability of the equipment and four of which related to ancillary services. Because this study targeted passive ambient sensing, these items were removed, and informational safety (security and consideration for privacy), integration with the indoor environment (unobtrusiveness of the sensor), no change in usual life in the home, and good exterior design (shape and color) were included as alternative items.

## 4. Results

The scores for health status, self-efficacy, and the JST version of the activity competence index are shown in Table 2. The median subjective health status was 3 (normal), with a mean of 2.53 (SD = 1.00). The median self-efficacy was 29, with a mean of 28.83 (SD = 5.76). The median value for the JST version of the activity competence index was 11, with a mean of 11.01 (SD = 2.79).

### 4.1. The Factors That Would Encourage Respondents to Install Sensors

As for the factors that would encourage older people to install sensors in the home, the medians for “reduced expenses” and “increased income” were “3: Slightly applicable”, while the medians for the other uses were “4: Neither” (Table 3). Referring to the averages comparing factors that encourage installation, the most important was 3.44 (SD = 1.47) for increased income, and the lowest was 4.00 (SD = 1.28) for social participation.

Increasing income, reducing expenditures, improving health, autonomous living support, maintaining health, maintaining mental health, improving mental health, and social participation were the factors that older persons chose as influencing acceptance of installation of sensors in their homes. While many studies on sensors for older people have discussed improved health care and avoidance of future illness as objectives, the results showed that, at least from the perspective of current users, financial gain and avoidance of loss motivate acceptance more than the maintenance and improvement of physical and mental health.

### 4.2. The Types of Sensors That Face Resistance

The medians for cameras and microphones were both rated as “5: Slightly resist”, and conversely, doors/windows, temperature/humidity, CO_2_/gas/smoke, and water flow were “3: Slightly not resist”, and the other sensor types were “4: Neither” (Table 4). Referring to the averages to make resistance-to-installation comparisons according to sensor type, the highest resistance was 4.83 (SD = 1.64) for microphones, and the lowest was 2.87 (SD = 1.50) for CO_2_, gas, and smoke sensors.

Thus, the sensors to which the elderly were resistant with respect to installation in their homes were microphones, cameras, interior walls/ceilings, floors/mats, temperature sensors, doors and windows, current, temperature/humidity, water flow, and CO_2_/gas/smoke, and they were rejected in that order.

### 4.3. The Important Factors Regarding Introduction of Sensors in the Home

The medians for “informational security” and “constancy of life” were “5: Very important”, and for “design quality” was “3: Neither”, while the medians for the other factors were “4: Slightly important” (Table 5). When referring to the means when comparing factors important for implementation, the most important factors were information security at 4.44 (SD = 0.90) and stability of life at 4.44 (SD = 0.91), and the least was design quality at 3.43 (SD = 1.05).

Thus, the factors that older adults considered important with regard to the installation of sensors in their homes and that were found to be supportive were informational safety, constancy of life, effectiveness, maintenance, physical safety, after-sales service, environmental integration, durability, ease of procedure, size, and design quality, in that order. Informational security, constancy of life, durability, effectiveness based on expectations, repair and maintenance services, physical safety, after-sales service, professional guidance and advice, environmental integration, procedures and time frames for obtaining, size, and design quality, in that order, were found to be important factors that influenced older people in their choice of sensors.

## 5. Analysis

### 5.1. Differences in Means of Health Status, Self-Efficacy, and Activity Capacity Indices in Basic Attributes of Participants

We compared the mean differences in health status, self-efficacy, and activity performance indicators across basic attributes such as gender, age, health status, self-efficacy, and activity performance indicators (Table 2). In this section, we analyze the results of differences in means (*t*-test) for each item (Table 6).

#### 5.1.1. Gender Differences

First, we analyzed the relationship between health status, self-efficacy, and activity performance indicators across gender. The mean of men’s health status was 2.51 (SD = 0.97), the mean of self-efficacy was 29.13 (SD = 5.67), and the mean of the activity capacity index was 10.74 (SD = 2.75), while the mean of women’s health status was 2.56 (SD = 1.03), the mean of self-efficacy was 28.53 (SD = 5.85), and the activity capacity index mean was 11.29 (SD = 2.81).

A test of the difference between the means of two samples assumed to have variances (*t*-test) for gender differences revealed a significant difference in the activity ability index. In the present study sample, women were found to maintain higher activity capacity index scores than men.

#### 5.1.2. Age Differences

Next, we compared the relationship between health status, self-efficacy, and activity capacity indices in terms of age. The means of health status, self-efficacy, and activity ability index for those aged 74 and below were 2.43 (SD = 1.02), 28.64 (SD = 5.77), and 10.74 (SD = 2.66), respectively, while the means of health status and self-efficacy for those aged 75 and above were 2.64 (SD = 0.97) and 29.03 (SD = 5.76), and the mean of the activity ability index was 11.29 (SD = 2.91). A t-test of the difference between the means of the two samples, which were assumed to have variances in terms of age, revealed a significant difference (*p* < 0.05) in the subjective health assessment and the activity ability index. In the current survey sample, it was found that the older age group’s subjective health was worse compared to the younger group, whereas the older age group maintained higher activity capacity index scores.

#### 5.1.3. Household Composition

The relationship between health status, self-efficacy, and activity capacity indices in household composition was compared. The means of health status, self-efficacy, and activity capacity index for older adults living alone were 2.54 (SD = 1.06), 28.95 (SD = 6.10), and 10.74 (SD = 2.79), respectively, while the means of health status and self-efficacy for older adults living in couples were 2.53 (SD = 0.95) and 28.71 (SD = 5.41), and their activity capacity index mean was 11.62 (SD = 2.67).

A test of the difference between the means of two samples assumed to have variances in household composition (*t*-test) revealed a significant difference (*p* < 0.05) in the activity ability index. In the present study sample, the elderly-couple households were found to maintain higher activity capacity indices.

#### 5.1.4. Subjective Health Assessment

We also compared the relationship between self-efficacy and activity capacity indices in health status.

The mean self-efficacy of the elderly in good health was 30.83 (SD = 5.29), and their mean activity capacity index was 11.30 (SD = 2.75), while the mean self-efficacy of the elderly in poor health was 26.99 (SD = 5.57), and their mean activity capacity index was 10.74 (SD = 2.81). The mean of the activity ability index was 10.74 (SD = 2.81).

In health status, a test of the difference between the means of two samples assumed to have variances (*t*-test) revealed significant differences in self-efficacy and activity ability indicators (*p* < 0.01). In the present study sample, we found that older adults in better health maintained higher self-efficacy and activity ability indices.

#### 5.1.5. Self-Efficacy

Furthermore, we compared the association between self-efficacy and activity ability indices in self-efficacy.

The mean health status of the older adults with high self-efficacy was 2.24 (SD = 0.95), and the mean activity capacity index was 11.65 (SD = 2.66), while the mean health status of the older adults with low self-efficacy was 2.82 (SD = 0.97), and the mean activity capacity index was 10.38 (SD = 2.78).

In self-efficacy, a test of the difference between the means of two samples assumed to have variances (*t*-test) revealed significant differences (*p* < 0.01) in health status and the activity ability index. In the present study sample, it was found that older adults with higher self-efficacy maintained higher health status and activity ability indices.

#### 5.1.6. Activity Capacity Index

The relationship between health status and self-efficacy in the activity capacity index is compared below. The mean of health status and self-efficacy of the elderly with high activity capacity index was 2.39 (SD = 0.94) and 30.65 (SD = 5.00), respectively, while the mean of health status and self-efficacy of the elderly with low activity capacity index was 2.65 (SD = 1.04) and 27.36 (SD = 5.93), respectively.

A test of the difference between the means of two samples assumed to have variances (*t*-test) was conducted on the activity ability index, and significant differences were found in health status and self-efficacy (*p* < 0.01). In the present study sample, it was found that the elderly with higher activity capacity indices maintained higher health status and self-efficacy.

**Table 6:** Summarizes the characteristics and responses of the participants in this study. Self-health assessment (score range 1–5, good is 1), self-efficacy (score range 10–40, best is 40), JST version activity capacity index (score range 0–16, best is 16).

Category	Number of Observation	Subjective Health Assessment				Self-Efficacy				Activity Capacity Index			
		Mean	SD	*p*-Value		Mean	SD	*p*-Value		Mean	SD	*p*-Value	
Men	200	2.51	0.97	0.583	n.s.	29.13	5.67	0.298	n.s.	10.74	2.75	**0.049**	*
Women	200	2.56	1.03			28.53	5.85			11.29	2.81		
Age 65–74	200	2.43	1.02	**0.032**	*	28.64	5.77	0.499	n.s.	10.74	2.66	**0.049**	*
Age over 75	200	2.64	0.97			29.03	5.76			11.29	2.91		
Living alone	200	2.54	1.06	0.960	n.s.	28.95	6.10	0.678	n.s.	10.41	2.79	**0.000**	**
Couple	200	2.53	0.95			28.71	5.41			11.62	2.67		
Self-health good	192					30.83	5.29	**0.000**	**	11.30	2.75	**0.044**	**
Self-health not good	208					26.99	5.57			10.74	2.81		
High Self-efficacy	197	2.24	0.95	**0.000**	**					11.65	2.66	**0.000**	**
Low Self-efficacy	203	2.82	0.97							10.38	2.78		
High Activity index	179	2.39	0.94	**0.007**	**	30.65	5.00	**0.000**	**				
Low Activity index	221	2.65	1.04			27.36	5.93						

(** *p* < 0.01, * *p* < 0.05).

### 5.2. Comparison of Differences in Introductory Use against Participant Attributes

In this section, we stated the results of a U-test conducted to analyze whether factors that promote sensor adoption differ by respondent attributes. The analyses were compared by gender, age, household composition, subjective health rating (high/low groups), self-efficacy (high/low groups), and activity capacity index (high/low groups), respectively (Table 7).

#### 5.2.1. Gender Differences

Mann–Whitney U-tests were conducted for 200 males and 200 females to gauge their attitudes toward acceptance of sensors. No significant differences were found between them for any of the parameters. This indicates that, among the older population in Japan, there are no significant differences between males and females in their attitude toward introduction of sensors.

#### 5.2.2. Household Composition

In addition, among the 200 older households that include living alone and among the 200 elderly-couple-only households, the Mann–Whitney U-test showed that there were no significant differences between the two groups in terms of the attitudes toward introduction of the sensor systems for any of the factors assessed. Of course, it is possible that there may be some differences in the parameters that promote acceptance among households, including those where children are living with the older people.

#### 5.2.3. Subjective Health Assessment

Regarding subjective health, 70 respondents (17.5%) answered “Good”, 122 (30.5%) answered “fair”, 138 (34.5%) answered “Normal”, 65 (16.3%) answered “Not so good”, and 5 (1.3%) answered “Poor”. In addition, a Mann–Whitney U-test was conducted for the attitudes of the 192 respondents who answered “Good” or “Fairly good” and the 208 who answered “Fair”, “Fairly poor”, or “Not good” on the subjective health evaluation. No significant differences were found in any of the factors assessed.

#### 5.2.4. Age Differences

Mann–Whitney U-tests were conducted for the 200 participants aged 65 to 74 years old and the 200 participants aged ≥75 years old to compare their responses to the different factors assessed.

The *p*-values (probability of significance) for health maintenance, health improvement, mental health improvement, social participation, and mental health maintenance were <0.01; the *p* values (probability of significance) for mental improvement and social participation were <0.05. No significant differences were found for other parameters.

The respective averages for the older group aged 65 to 74 years were lower than those for those aged ≥75 years, indicating a trend toward greater willingness to adopt the systems at home. It is thought that, as age increases, the desire to maintain and improve physical and mental health becomes stronger, which may lead to a willingness to install sensors.

#### 5.2.5. Self-Efficacy

Mann–Whitney U-tests were conducted for 197 participants with a total self-efficacy score of ≥30 and 203 participants with a total self-efficacy score of ≤29 to assess the difference between the groups in terms of acceptance of the sensors; *p*-values were <0.01 for mental maintenance, mental improvement, expenditure reduction, income increase, and autonomous living support, and no significant differences were found for the other parameters.

The mean value for the group of older people with a total self-efficacy score of 30 or higher indicated that they tended to be more willing to introduce sensors into their homes if they expected mental health improvement and maintenance, compared to the group with a total self-efficacy score of 29 or lower. Conversely, older people with a total self-efficacy score of 29 or lower tended to be more willing to introduce sensors into their homes if they expected expenditure reductions, income increases, and autonomous living support, compared to older people with a total self-efficacy score of 30 or higher.

#### 5.2.6. Activity Capacity Index

Mann–Whitney U-tests were conducted for the 179 participants with a total of ≥12 points on the activity capacity index and the 221 participants with ≤11 points on the activity capacity index, to assess the differences in their acceptance of sensors in their homes. The *p*-values were <0.01 for autonomous living support and <0.05.for mental health maintenance. No significant differences were found for the other factors.

The mean value of the group with high total scores on the activity capacity index was higher than that of the group with low scores. The results showed that the elderly group with higher scores on the activity ability index tended to be less willing to install the system in their homes. This suggests that the group with lower total scores on the activity capacity index was more willing to introduce the system for mental health maintenance and for autonomous living support in their homes.

## 6. Discussion

### 6.1. Mutual Relationships of Participants’ Subjective Health Ratings, Self-Efficacy, and Activity Capacity Index

When we tested for differences in means for the participants’ basic attributes of subjective health evaluation, self-efficacy, and activity ability index, we found significant differences for all items. In other words, high subjective health evaluation, high self-efficacy, and high activity ability index are considered to be related.

### 6.2. Comparison of the Paper’s Sample with the Japanese Average

**Figure 1:**
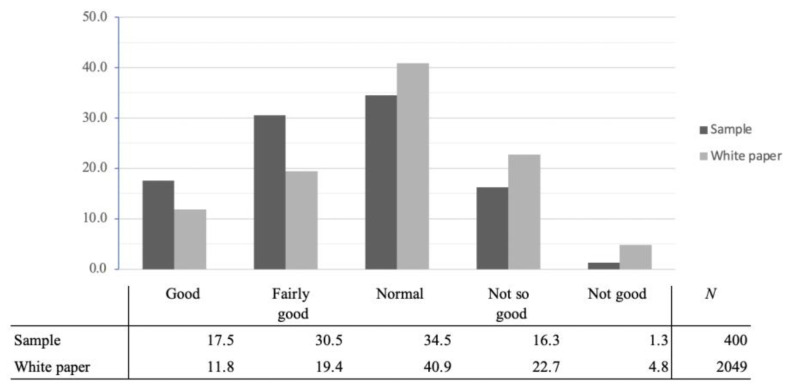
Comparison of survey sample and government white paper on subjective health assessment.

With regard to the subjective health status of the over-65s in the government paper, 11.8% responded “Good”, 19.4% responded “Fair to good”, 40.9% responded “Normal”, 22.7% responded “Not so good”, and 4.8% responded “Not good” (Figure 1). Compared to the percentages in the current sample, more respondents answered “Good” or “Fairly good” in the current sample, and conversely, more respondents answered “Normal”, “Not so good”, or “Not good” in the government paper. This indicates the possibility that a slightly healthier group was extracted compared to the national average, since those participating were willing to voluntarily complete the survey.

**Figure 2:**
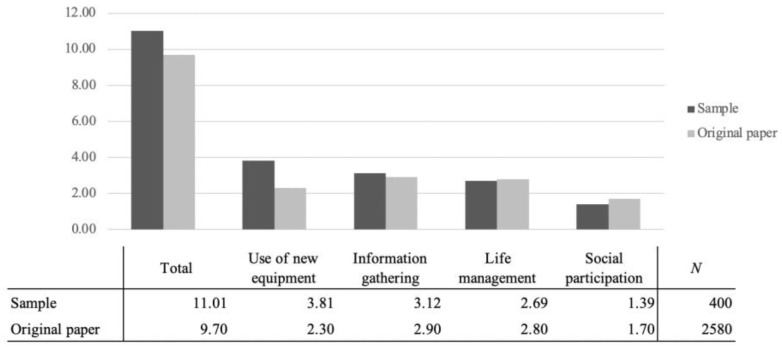
Comparison of survey sample and previous study on activity capacity index.

With regard to the activity capacity index, the sample tended to have a higher use of information equipment compared to the national average from the previous study (Figure 2). This may be due to the fact that this survey was conducted using an online questionnaire, which requires the use of a PC or tablet to answer the questions, which may have contributed to the higher use of information devices. No significant differences were found for the other items.

On the other hand, according to the Cabinet Office (2020) “Public Opinion Survey on the Use of Information and Communication Devices”, the total number of respondents who answered that they “often” or “sometimes” use smartphones and tablets was 77.8% for the population as a whole. By age, however, the usage rate for the 18–29 age group was nearly 100%, at 98.7%, while the usage rate declined as age increased, with 73.4% for the 60–69 age group and only 40.8% for the 70+ age group. Therefore, it is necessary to interpret the results of this study with the understanding that the study was conducted among older adults who are able to use the internet.

### 6.3. Factors Influencing the Uses for Sensor Adoption

The results of a U-test indicated that there were three factors that influenced the adoption of sensors: age, activity capacity index, and self-efficacy. First, for age differences, the group of elderly aged 75 years and older tended to be significantly more willing to install the sensors in their homes if they were expected to maintain health, improve health, maintain mental health, improve mental health, and participate in society, compared to the group of the elderly aged 65 to 74 years. It is thought that as age increases, people have stronger ideas about maintaining and improving their physical and mental health, which may lead to their willingness to introduce sensors.

Next, the group with a higher total score on the activity ability index (12 points or higher) tended to be more motivated to introduce sensors for mental maintenance, autonomous living support, and social participation than the group with a lower total score (11 points or lower). It is thought that they may be more motivated to adopt the program for information that helps them to lead autonomous lives, with an emphasis on mental health maintenance and social participation.

The group of older people with a total self-efficacy score of 30 or higher tended to be more willing to introduce sensors into their homes if it was expected to improve and maintain mental health, compared to the group with a total self-efficacy score of 29 or lower. Conversely, older people with a total self-efficacy score of 29 or lower tended to be more willing to introduce sensors into their homes if they expected expenditure reductions, income increases, and autonomous living support, compared to older people with a total self-efficacy score of 30 or higher. This indicated the possibility that factors that increased willingness to introduce the systems differed depending on the level of self-efficacy.

Much of the research on support devices (robots, IoT devices, and sensors) for the elderly often proposes one form of outreach to single-person or couple-only households with limited social participation. The results of this study showed that information support and social participation can facilitate the adoption of sensors by older people with low activity capacity indicators in order that they lead autonomous lives. Social participation may help maintain and improve mental health and detect abnormalities; however, it requires a user interface to provide information in their homes. For example, a previous study has proposed human–robot interaction systems in which small robots encourage users to go out and exercise during the day when detected activity is low based on sensors installed in the house [38]. Such a functionality could be useful in developing a system that is tailored to the older user’s intentions, as it not only encourages the user’s social participation, but also allows for interventions that increase outings and exercise.

### 6.4. Relational Model

**Figure 3:**
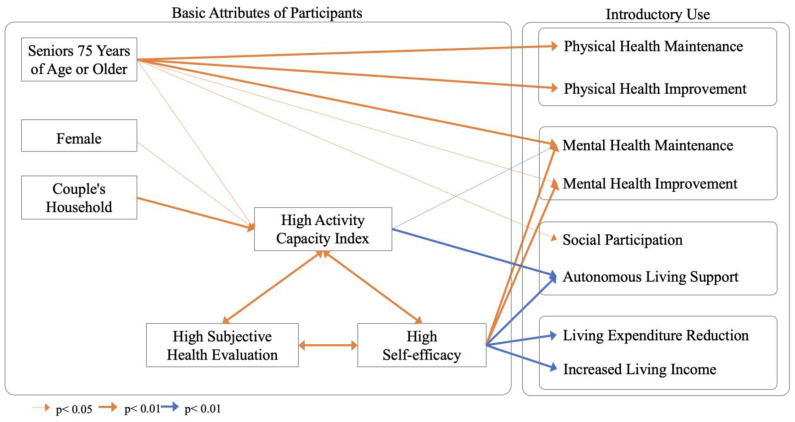
The relationship of the basic attributes of the participants to use of sensors. Red lines indicate increasing impacts; blue lines indicate decreasing impacts.

Section 6.1 and Section 6.2 are organized according to cognitive model, as shown in Figure 3. Higher and lower age are related to physical and mental health and social participation as independent indicators. In other words, older age groups are more likely to seek physical and mental health and social participation, and they therefore are more likely to adopt sensors that are expected to be effective for these applications. In addition, high- and low-activity capacity indices are related to social participation and information benefits. In other words, people with high activity ability index scores are more likely to seek information related to social participation and leading an autonomous life, and thus they are more likely to adopt sensors that are expected to be effective for this purpose.

And the activity ability index and subjective health evaluation scores affect high and low self-efficacy. The group with low self-efficacy will be more likely to seek information benefits, maintenance of mental health, and financial benefits (increased income and reduced expenses). Therefore, it is thought that those with low self-efficacy will be more likely to adopt sensors that are expected to be effective for their use. The elderly who are likely to need sensors in the future also have various attributes, and the introduction of ambient sensors in elderly households may be further advanced by recommending applications that are easy to introduce based on the attributes of the target population, rather than discussing all of them in general.

## 7. Limitations

This online survey was designed to have a sample size that would be highly representative of the older people living in communities in Japan. By designing the survey as an online survey, equal allocation to attributes such as gender, age group, and residential type (single or married-couple-only) was made possible, allowing for a variety of analyses.

Furthermore, according to Japan’s Ministry of Internal Affairs and Communications “Telecommunication Usage Trends Survey”, the internet usage rate by age group is 84.4% for those aged 60–69, 59.4% for those aged 70–79, and 27.6% for those aged 80 and older [39]. Therefore, since the survey respondents were among those who used PCs and smartphones on a regular basis, it is conceivable that differences in IT literacy could affect their acceptance of installing sensors in their homes. Further research is required to understand the impact of IT literacy.

## 8. Conclusions

For this study, an online survey was conducted to discover the preferences of older adults regarding sensor use in their households, rather than relying on the preferences of the researchers who develop them. The sample size was 400 Japanese community-dwelling people aged 65 years and older. In the sample, men and women, single-person and couple-only households, and younger seniors (younger than 74 years old) and older seniors (older than 75 years old) were assigned in the same numbers.

When we tested for differences in means for the participants’ basic attributes of subjective health evaluation, self-efficacy, and activity ability, we found significant differences for all items. In other words, high subjective health evaluation, high self-efficacy, and high activity ability index scores are considered to be related. As for the activity capacity index used with our sample, the sample tended to have higher use of information equipment compared to the national average from the previous study. This may be due to the fact that this survey was conducted using an online questionnaire, which requires the use of a PC or tablet to answer the questions, which may have contributed to the higher use of information devices. Therefore, it is necessary to interpret the results of this study with the understanding that the study was conducted among older adults who are able to use the internet.

As for the factors that would encourage older people to install sensors in the home, the survey results showed that “reduced expenses” and “increased income” were rated highly in importance while physical and mental improvements were rated as less important.

Looking at the results regarding the type of sensors that face resistance, we found that both “cameras” and “microphones” were evaluated as facing slightly strong resistance, while “door/window”, “temperature/humidity”, “CO_2_/gas/smoke”, and “water flow” were evaluated as not facing such strong resistance. Furthermore, regarding factors important to the introduction of sensors in the home, “informational security” and “constancy of life” were considered more important than other factors for installing sensors, while design quality was rated the least important.

The results of our analysis indicated that the factors that entail the installation of sensors may differ by age, gender, and household composition. The older adults who are likely to need sensors in the future also have various attributes, and the introduction of ambient sensors in their households may be further advanced by recommending applications that are easy to introduce based on the attributes of the target population, rather than by discussing all of them in general.

## Figures and Tables

**Table 1:** Questionnaire items.

**[Subjective health assessment]** □ Please select the most applicable answer to the following questions below. What is your current subjective health status? (Choose one.) 1 Good 2 Fairly good 3 Normal 4 Not so good 5 Not good
**[Self efficacy]** □ Please select the most applicable answer to the following questions below. Answer list: 1 Not at all true 2 Not very true 3 Somewhat true 4 True 2. If I work hard, I can solve difficult problems. 3. Even if someone disagrees with me, I can find ways and means to get what I want. 4. It is not difficult for me to achieve my goals without losing sight of them. 5. I am confident that I can deal effectively with unexpected events. 6. I have the ability to be resourceful and know how to deal with unforeseen situations. 7. With the necessary effort, I can solve most problems. 8. I believe in my ability to cope, so I can remain calm in the face of challenges. 9. When faced with a problem, I can come up with several solutions. 10. When I am in difficulty, I can come up with a solution. 11. No matter what happens, I can handle it.
**[Activity capacity index]** □ Please tell us about your daily life. Answer list: 1. yes 2. no 12. Do you use a cell phone? 13. Do you use an ATM? 14. Do you operate a video or DVD player? 15. Do you use a cell phone or computer for e-mail? 16. Are you interested in foreign news and events? 17. Can you judge the credibility of health information? 18. Do you appreciate art, movies, and music? 19. Do you watch educational programs 1. yes 2. no 20. Do you take measures to avoid becoming a victim of fraud, snatching, burglary, etc.? 21. Do you make small efforts in your daily life? 22. Are you able to take care of the sick? 23. Do you care for your grandchildren, family members, or acquaintances? 24. Do you participate in local festivals and events? 25. Are you active in neighborhood associations? 26. Are you willing to take on the role of a caretaker or a position in a neighborhood association or group activity? 27. Are you involved in service activities or volunteer work?
**[The factors that would encourage respondents to install sensors]** □ Please tell us if you would be willing to install a sensor in your home to assist you in your daily life if you could expect the following benefits: The sensors we are discussing here are not for you, but for your living environment. Answer list: 1. Extremely applicable 2. Moderately applicable 3. Slightly applicable 4. Neither 5. Slightly not applicable 6. Moderately not applicable 7. Extremely not applicable 28. If you expect to maintain your current physical health (and avoid future illness/disability), would you be willing to install sensors in your home to assist you in your daily life? 29. If you expect to enhance your current physical health (improve your current situation), please tell me if you would be willing to install sensors in your home to support your daily life. 30. If you expect to maintain your current mental health (avoid future illness/disability such as depression or cognitive decline), please tell me if you would be willing to install sensors in your home to support your daily life. 31. If you expect to improve your current mental health (improve current conditions such as depression or cognitive decline), please tell me if you would be willing to install sensors in your home to assist you in your daily life. 32. If you expect to reduce your current financial expenses (spend less), please tell me if you would be willing to install sensors in your home to assist you in your daily life. 33. If you expect to increase your current financial income (increase your income), please tell me if you would be willing to install sensors in your home to support your lifestyle. 34. If you expect to receive informational benefits related to autonomous living support (reminders about daily routines, medications, etc.), please tell me if you would be willing to install sensors in your home to assist you in your daily life. 35. If you expect to receive informational benefits related to your involvement with others and the community (social participation) (recommendations about community and government events), please tell us if you would be willing to install a sensor in your home to support your daily life.
**[Types of sensors that face resistance]** □ Please tell us how much you would resist installing the following types of sensors in your home: Answer list: 1. Extremely not resist 2. Moderately not resist 3. Slightly not resist 4. Neither 5. Slightly resist 6. Moderately resist 7. Extremely resist 36. Please tell us how much resistance you have to installing sensors on walls and ceilings to detect the movement of people. 37. Please tell me how much resistance you have to installing sensors on the floor or under mats to detect human movement. 38. How much resistance is there to installing sensors for opening/closing doors and windows to detect human movement? 39. How resistant are you to installing cameras to detect human movement? 40. How much resistance do you have to installing a microphone to detect biometric information? 41. Please tell me how much resistance you have to installing a body temperature sensor to obtain biometric information. 42. Please tell me how much resistance you have to installing a temperature/humidity sensor to understand your living environment. 43. Please tell me how much resistance you have to installing Co_2_, gas, and smoke sensors to understand your living environment. 44. Please tell me how much resistance you have to installing current sensors to understand your living environment. 45. Please tell me how much resistance you have to installing water-flow sensors to understand your living conditions.
**[Necessary elements to consider when installing sensors in your home]** □ Please tell me how important the following items are for the installation of sensors in your home: Answer list: 1. Not important at all 2. Not very important 3. Neither 4. Slightly important 5. Very important 46. Size (height, length, width) 47. Physical safety 48. Durability (sturdiness) 49. Effectiveness based on your expectations 50. Procedures and time frames for obtaining sensors 51. Repair and maintenance services 52. Professional guidance and advice (e.g., information) 53. After-sales service 54. Informational security (safety of personal information, privacy considerations) 55. Constancy of life (no change in normal life at home) 56. Environmental integration (unobtrusive sensors) 57. Design quality (external shape and color)

**Table 2:** Median, mean, and SD of self-health assessment (score range 1–5, good is 1), self-efficacy (score range 10–40, best is 40), JST version Activity Capacity Index (score range 0–16, best is 16) in the sample.

	Self-Health Assessment	Self-Efficacy	Activity Capacity Index
Median	3	29	11
Mean	2.53	28.83	11.01
SD	1.00	5.76	2.79

**Table 3:** Median, mean, and SD of the factors that would encourage people to install sensors. Answer list: 1. Extremely applicable 2. Moderately applicable 3. Slightly applicable 4. Neither 5. Slightly not applicable 6. Moderately not applicable 7. Extremely not applicable.

No.	Content	Median	Mean	SD
28	Physical maintenance	4	3.67	1.38
29	Physical improvement	4	3.55	1.34
30	Mental maintenance	4	3.89	1.45
31	Mental improvement	4	3.92	1.47
32	Expenditure reduction	3	3.46	1.44
33	Income increase	3	3.44	1.47
34	Autonomous living support	4	3.66	1.3
35	Social participation	4	4.00	1.28

**Table 4:** Median, mean, and SD of resistive sensor type for older people. Answer list: 1. Extremely not resist 2. Moderately not resist 3. Slightly not resist 4. Neither 5. Slightly resist 6. Moderately resist 7. Extremely resist.

No.	Content	Median	Mean	SD
36	Interior walls/ceilings	4	4.05	1.77
37	Floors/mats	4	3.99	1.74
38	Doors and windows	3	3.56	1.74
39	Camera	5	4.79	1.69
40	Microphone	5	4.83	1.64
41	Body temperature	4	3.66	1.64
42	Temperature/humidity	3	3.33	1.62
43	CO_2_, gas, smoke	3	2.87	1.50
44	Current	4	3.46	1.59
45	Water flow	3	3.20	1.57

**Table 5:** Median, mean, and SD of product features that influenced older people in their acceptance of sensors. Answer list: 1. Not important at all 2. Not very important 3. Neither 4. Slightly important 5. Very important.

No.	Content	Median	Mean	SD
46	Size (height, length, width)	4	3.48	1.08
47	Physical safety	4	4.14	1.07
48	Durability (Sturdiness)	4	3.80	1.03
49	Effectiveness for your expectations	4	4.24	0.95
50	Procedures and time frame for obtaining	4	3.61	1.01
51	Repair and maintenance services	4	4.16	0.92
52	Professional guidance and advice	4	3.80	0.96
53	After-sales service	4	4.11	0.92
54	Infomational security	5	4.44	0.90
55	Constancy of life	5	4.44	0.91
56	Environmental integration	4	3.80	1.02
57	Design quality	3	3.43	1.05

**Table 7:** Summarizes the characteristics and responses of the participants in this study.

Category	Number of Observation	Introductory Use	Mean for Sample	Mean for Group 1	Mean for Group 2	U-Value	*p*-Value	
Gender differences	Men = 200 (Group 1) Women = 200 (Group 2)	Physical maintenance	3.67	3.70	3.64	19,500	0.655	n.s.
		Physical improvement	3.55	3.57	3.53	19,192	0.471	n.s.
		Mental maintenance	3.89	3.91	3.87	19,611	0.728	n.s.
		Mental improvement	3.92	4.00	3.84	19,118	0.433	n.s.
		Expenditure reduction	3.46	3.37	3.54	18,684	0.240	n.s.
		Income increase	3.44	3.32	3.57	18,020	0.079	n.s.
		Autonomous living support	3.66	3.65	3.68	19,343	0.554	n.s.
		Social participation	4.00	4.04	3.96	19,622	0.733	n.s.
Age Differences	Age 65–74 = 200 (Group 1) Age over 75 = 200 (Group 2)	**Physical maintenance**	3.67	**3.89**	**3.45**	**16,190**	**0.001**	**
		**Physical improvement**	3.55	**3.72**	**3.38**	**16,942**	**0.006**	**
		**Mental maintenance**	3.89	**4.08**	**3.70**	**16,818**	**0.005**	**
		**Mental improvement**	3.92	**4.10**	**3.74**	**17,104**	**0.010**	*
		Expenditure reduction	3.46	3.48	3.43	19,242	0.499	n.s.
		Income increase	3.44	3.44	3.45	19,930	0.950	n.s.
		Autonomous living support	3.66	3.78	3.55	18,131	0.092	n.s.
		**Social participation**	4.00	4.15	3.86	17,316	0.016	*
Household Composition	Living alone = 200 (Group 1) Living couple = 200 (Group 2)	Physical maintenance	3.67	3.64	3.70	19,636	0.745	n.s.
		Physical improvement	3.55	3.52	3.58	19,536	0.678	n.s.
		Mental maintenance	3.89	3.86	3.92	19,767	0.835	n.s.
		Mental improvement	3.92	3.96	3.89	19,137	0.443	n.s.
		Expenditure reduction	3.46	3.31	3.60	17,932	0.065	n.s.
		Income increase	3.44	3.31	3.58	18,000	0.076	n.s.
		Autonomous living support	3.66	3.68	3.65	19,739	0.814	n.s.
		Social participation	4.00	4.09	3.92	18,408	0.151	n.s.
Self-efficacy	High Self-efficacy groups 30 points or more = 197 (Group 1) Low Self-efficacy group 29 points or less = 203 (Group2)	Physical maintenance	3.67	3.66	3.67	18,261	0.122	n.s.
		Physical improvement	3.55	3.51	3.58	18,058	0.083	n.s.
		**Mental maintenance**	3.89	**3.86**	**3.91**	**16,881**	**0.005**	**
		**Mental improvement**	3.92	**3.91**	**3.93**	**16,579**	**0.002**	**
		**Expenditure reduction**	3.46	**3.47**	**3.44**	**16,702**	**0.003**	**
		**Income increase**	3.44	**3.47**	**3.42**	**16,861**	**0.005**	**
		**Autonomous living support**	3.66	**3.68**	**3.64**	**16,756**	**0.004**	**
		Social participation	4.00	3.99	4.00	19,036	0.387	n.s.
Activity Capacity Index	High Activity index groups 12 points or more = 179 (Group 1) Low Activity index group 11 points or less = 221 (Group 2)	Physical maintenance	3.67	3.76	3.58	18,527	0.261	n.s.
		Physical improvement	3.55	3.65	3.44	17,863	0.084	n.s.
		**Mental maintenance**	3.89	**4.10**	**3.68**	**17,410**	**0.034**	*
		Mental improvement	3.92	4.15	3.70	17,742	0.069	n.s.
		Expenditure reduction	3.46	3.69	3.23	19,770	0.993	n.s.
		Income increase	3.44	3.66	3.23	19,685	0.932	n.s.
		**Autonomous living support**	3.66	**3.85**	**3.48**	**16,912**	**0.009**	**
		Social participation	4.00	4.08	3.92	13,448	9.627	n.s.
Subjective Health Assessment	Self-health good group answered “1 or 2” = 192 (Group 1) Self-health not good group answered “3 or 4 or 5” = 208 (Group 2)	Physical maintenance	3.67	3.58	3.74	19,895	0.948	n.s.
		Physical improvement	3.55	3.42	3.65	19,390	0.605	n.s.
		Mental maintenance	3.89	3.74	4.01	19,515	0.686	n.s.
		Mental improvement	3.92	3.79	4.03	19,709	0.818	n.s.
		Expenditure reduction	3.46	3.45	3.46	19,529	0.695	n.s.
		Income increase	3.44	3.45	3.43	19,758	0.852	n.s.
		Autonomous living support	3.66	3.49	3.80	19,584	0.729	n.s.
		Social participation	4.00	3.61	4.32	19,806	0.884	n.s.

(** *p* < 0.01, * *p* < 0.05).

## Data Availability

The data that support the findings of this study are available from the corresponding authors upon reasonable request.
